# Transforming growth factor beta (TGF-β) in adolescent chronic fatigue syndrome

**DOI:** 10.1186/s12967-017-1350-1

**Published:** 2017-12-04

**Authors:** Vegard Bruun Wyller, Chinh Bkrong Nguyen, Judith Anita Ludviksen, Tom Eirik Mollnes

**Affiliations:** 1 Department of Pediatrics and Adolescent Health, Akershus University Hospital, 1478 Lørenskog, Norway; 2 Division of Medicine and Laboratory Sciences, University of Oslo, Oslo, Norway; 3 Research Laboratory, Nordland Hospital, Bodø, Norway; 4 Faculty of Health Sciences, K.G. Jebsen TREC, University of Tromsø, Tromsø, Norway; 5 Department of Immunology, Oslo University Hospital, Oslo, Norway; 6 K.G. Jebsen IRC, University of Oslo, Oslo, Norway; 7 Centre of Molecular Inflammation Research, Norwegian University of Science and Technology, Trondheim, Norway

**Keywords:** Chronic fatigue syndrome, Adolescent, Inflammation, Transforming growth factor beta

## Abstract

**Background:**

Chronic fatigue syndrome (CFS) is a prevalent and disabling condition among adolescent. The disease mechanisms are unknown. Previous studies have suggested elevated plasma levels of several cytokines, but a recent meta-analysis of 38 articles found that of 77 different cytokines measured in plasma, transforming growth factor beta (TGF-β) was the only one that was elevated in patients compared to controls in a sufficient number of articles. In the present study we therefore compared the plasma levels of the three TGF-β isoforms in adolescent CFS patients and healthy controls. In addition, the study explored associations between TGF-β levels, neuroendocrine markers, clinical markers and differentially expressed genes within the CFS group.

**Methods:**

CFS patients aged 12–18 years (n = 120) were recruited nation-wide to a single referral center as part of the NorCAPITAL project (ClinicalTrials ID: NCT01040429). A broad case definition of CFS was applied, requiring 3 months of unexplained, disabling chronic/relapsing fatigue of new onset, whereas no accompanying symptoms were necessary. Healthy controls (n = 68) were recruited from local schools. The three isoforms of TGF-β (TGF-β1, TGF-β2, TGF-β3) were assayed using multiplex technology. Neuroendocrine markers encompassed plasma and urine levels of catecholamines and cortisol, as well as heart rate variability indices. Clinical markers consisted of questionnaire scores for symptoms of post-exertional malaise, inflammation, fatigue, depression and trait anxiety, as well as activity recordings. Whole blood gene expression was assessed by RNA sequencing in a subgroup of patients (n = 29) and controls (n = 18).

**Results:**

Plasma levels of all three isoforms of TGF-β were equal in the CFS patients and the healthy controls. Subgrouping according to the Fukuda and Canada 2003 criteria of CFS did not reveal differential results. Within the CFS group, all isoforms of TGF-β were associated with plasma cortisol, urine norepinephrine and urine epinephrine, and this association pattern was related to fatigue score. Also, TGF-β3 was related to expression of the B cell annotated genes *TNFRSF13C* and *CXCR5.*

**Conclusions:**

Plasma levels of all TGF-β isoforms were not altered in adolescent CFS. However, the TGF-β isoforms were associated with neuroendocrine markers, an association related to fatigue score. Furthermore, TGF-β3 might partly mediate an association between plasma cortisol and B cell gene expression.

*Trial registration* Clinical Trials NCT01040429

**Electronic supplementary material:**

The online version of this article (10.1186/s12967-017-1350-1) contains supplementary material, which is available to authorized users.

## Background

Chronic fatigue syndrome (CFS) is a long-lasting and disabling condition characterized by disproportional fatigue after exertions, musculoskeletal pain, headaches, cognitive impairments, orthostatic intolerance and other symptoms [1, 2]. CFS is a major threat towards adolescent health. The prevalence among teenagers is estimated at 0.1–1.0% [3, 4], and the condition may have detrimental effects on psychosocial and academic development [5], as well as family functioning [6].

Disease mechanisms of CFS remain poorly understood. However, several studies indicate modest immunological alterations, such as enhancement of systemic inflammation [7–9] and attenuation of B cell function [10–12]. A recent study on whole blood gene expression in CFS corroborated a general picture of increased innate immunity and decreased B cell differentiation and survival in CFS [13].

Despite these findings, studies of plasma cytokine levels have been inconclusive; findings include increased levels of interleukin (IL)-1 and tumor necrosis factor (TNF) [14], increased levels of IL-1α and IL-1β but normal levels of TNF [15], and no differences between CFS patients and healthy controls [16, 17]. A recent review including meta-analysis of 38 articles studying all together 77 different cytokines, including interleukins, chemokines and growth factors, found evidence of elevated blood levels only of transforming growth factor beta (TGF-β) in CFS (63% of the studies), whereas other cytokines were not found to be consistently altered in any direction [18]. In a previous study from our laboratory [17], we found that the 27 plasma cytokines measured were identical in the CFS and the control group, without any tendency for differences. This study using modern multiplex technique did not, however, include TGF-β, which here prompted us to investigate our population for this marker, in particular extending inclusion of all three different isoforms (denoted TGF-β1, TGF-β2 and TGF-β3).

TGF-β is excreted by macrophages and other immune cells. All isoforms are involved in a multitude of regulatory processes, including control of inflammation and specific immunity [19]. Notably, in some conditions, TGF-β promotes inflammatory responses combined with an inhibitory effect on several stages in B cell differentiation and survival. Thus, TGF-β is a possible mediator of the subtly skewed immune responses observed in a previous CFS study [13]. Furthermore, TGF-β stimulates the generation of regulatory T cells [20], which has been shown to be increased in CFS by previous reports [21, 22].

In addition to immune changes, CFS disease mechanisms are characterized by neuroendocrine alterations including enhanced sympathetic and attenuated parasympathetic cardiovascular nervous activity [23–26] and attenuation of the hypothalamus–pituitary–adrenal (HPA) axis [27–29]. These phenomena might be causally related, as suggested in the “sustained arousal” model of CFS [30]. For instance, sympathetic activity is shown to enhance TGF-β3 mRNA expression in macrophages [31]. Another study has demonstrated a relationship between HPA dynamics and plasma levels of TGF-β1 [32]. Recently, whole blood gene expression of immune annotated genes in CFS was demonstrated to be strongly associated with neuroendocrine markers of altered HPA-axis and autonomic nervous activity [13]. Thus, a possible relationship between neuroendocrine alterations and TGF-β levels in CFS deserves further attention.

Symptoms that might suggest immune activation, such as chills, sore throat and tender lymphatic nodes, are common complaints among CFS sufferers [1, 33]. A previous study revealed no associations between plasma cytokine levels and symptom scores [17]. Still, a relationship between differential gene expression and symptoms of post-exertional malaise, which is considered a hallmark of the CFS phenotype, has been observed [13]. This finding warrants further exploration of associations between clinical symptoms and immune markers, including TGF-β.

In general, the scientific investigation of adolescent CFS suffers several shortcomings as compared to adult CFS. For instance, to the best of our knowledge, no other studies have specifically addressed circulating TGF-β expression in adolescent CFS patients. Nevertheless, adolescent studies might benefit from low prevalence of comorbidity as well as a limited possibility of confounding effects from aging processes.

Taken together, the main aim of the present study was to compare plasma levels of TGF-β isoforms in adolescent CFS patients and healthy controls. We hypothesized increased levels among the CFS patients based on the previous meta-analysis [18]. In addition, the present study had two exploratory aims: (a) To explore associations between TGF-β levels, neuroendocrine markers and differentially expressed genes in the CFS group, and (b) To explore associations between TGF-β levels and CFS clinical symptoms.

## Methods

### CFS patients

This study is part of the NorCAPITAL-project (The Norwegian Study of Chronic Fatigue Syndrome in Adolescents: Pathophysiology and Intervention Trial; ClinicalTrials ID: NCT01040429). Details of the recruitment procedure and inclusion/exclusion criteria are described elsewhere [34]. Briefly, all hospital pediatric departments in Norway (n = 20), as well as primary care pediatricians and general practitioners, were invited to refer CFS patients aged 12–18 years consecutively to our study center. A standard form required the referral unit to confirm the result of clinical investigations considered compulsory to diagnose pediatric CFS according to national Norwegian recommendations (pediatric specialist assessment, comprehensive hematology and biochemistry analyses, chest X-ray, abdominal ultrasound, and brain MRI). Also, the referring units were required to confirm that the patient (a) was unable to follow normal school routines due to fatigue; (b) was not permanently bedridden; (c) did not have any concurrent medical or psychiatric disorder that might explain the fatigue; (d) did not experience any concurrent demanding life event (such as parents’ divorce) that might explain the fatigue; (e) did not use pharmaceuticals (including hormone contraceptives) regularly. Patients considered eligible to this study were summoned to a clinical encounter at our study center after which a final decision on inclusion was made. The CFS group comprised 120 patients.

In agreement with clinical guidelines [2] and previous studies from our group [24–26], we applied a ‘broad’ case definition of CFS, requiring 3 months of unexplained, disabling chronic/relapsing fatigue of new onset. We did not require that patients meet any other accompanying symptom criteria. Subgrouping of the participants according to the Fukuda case definition [35] and Canada 2003 case definition [33] was performed post hoc, based on questionnaire results (cf. below).

### Healthy controls

A group of healthy controls (n = 68) with a comparable distribution of gender and age were recruited from local schools. Controls were not matched to cases on any variable. No chronic disease and no regular use of pharmaceuticals (including hormone contraceptives) were allowed.

### Study design and ethics

A 1-day in-hospital assessment included clinical examination and blood sampling and always commenced between 7.30 and 9.30 a.m. All participants were instructed to fast overnight and abstain from tobacco products and caffeine for at least 48 h. The participants were instructed to apply an ointment containing the local anesthetic lidocaine (Emla^®^) on the skin in the antecubital area 1 h in advance. After at least 5 min supine rest in calm surroundings, blood samples were obtained in a fixed sequence from antecubital venous puncture. A questionnaire was completed after the clinical encounter and returned in a pre-stamped envelope.

Data were collected in the period from March 2010 until October 2012. The NorCAPITAL project has been approved by the Norwegian National Committee for Ethics in Medical Research (Reference ID: 2009/2541 S-09354c**)**. Written informed consent was obtained from all participants and from parents/next-of-kin if required. CFS patients were randomized to treatment with low-dose clonidine or placebo and followed for a total of 30 weeks; however, only baseline data are used in the present study. Details of the design are reported elsewhere [34].

### TGF-β isoforms: TGF-β1, TGF-β2 and TGF-β3

The blood samples for TGF-β analyses were collected in 4 ml EDTA tubes. The samples were placed on ice for approximately 30 min; thereafter, plasma was separated by centrifugation (2500*g*, 10 min, 4 °C) and frozen at − 80 °C until assayed. The samples were analyzed using Bio-Plex Human TGF-β 3-plex (Bio-Rad Laboratories Inc., Hercules, CA), containing the three isoforms of TGF-β: TGF-β1, TGF-β2 and TGF-β3. The assay was performed using the Bio-Plex 200 system (Bio-Rad Laboratories Inc.) according to the manufactures’ instructions. All samples were obtained, treated stored and analysed in exactly in the same way, using the same personnel at each step, to exclude methodological variation since TGFβ is sensible for sample handling, including release from platelets.

### Neuroendocrine markers

Blood samples for analyses of plasma norepinephrine and epinephrine were obtained in vacutainer tubes treated with ethylene glycol tetraacetic acid (EGTA)–glutathione. The samples were placed on ice for approximately 30 min, and thereafter separated by centrifugation (2250×*g*, 15 min, 4 °C) and assayed by high-performance liquid chromatography (HPLC) with a reversed-phase column and glassy carbon electrochemical detector (Antec, Leyden Deacade II SCC, Zoeterwoude, The Netherlands) as described in detail elsewhere [29]. Blood samples for analyses of plasma cortisol were obtained in 4 ml EDTA vacutainer tubes; plasma cortisol levels were determined by routine assays at the accredited laboratory at Oslo University Hospital, Norway. Morning spot urine samples for norepinephrine and epinephrine analyses were assayed with the same HPLC protocol as for plasma measurements, whereas morning spot urine free cortisol (non-conjugated cortisol) was assayed by solid phase competitive luminescence immunoassay (type Immulite^®^ 2000, Siemens Healthcare Diagnostics, NY, USA). The urine levels of creatinine were analyzed using standard automatic analyzer techniques at the accredited laboratory at Oslo University Hospital, Norway.

Indices of heart rate variability (HRV) were obtained from ECG recordings of participants laying in a horizontal position and connected to the Task Force Monitor (TFM) (Model 3040i, CNSystems Medizintechnik, Graz, Austria). Methodological details are provided elsewhere [36]. Power spectral analysis of HRV was automatically provided by the TFM, returning numerical values for low frequency (LF) power (0.05–0.17 Hz), high frequency (HF) power (0.17–0.4 Hz) and the LF/HF ratio. HF power is considered indicative of parasympathetic heart rate modulation, LF power reflects the combined effect of sympathetic and parasympathetic heart rate control, whereas the LF/HF ratio is an index of sympathetic/parasympathetic balance.

### Routine blood analyses

The serum concentration of C-reactive protein (CRP) was analyzed using a highly sensitive assay (Roche Diagnostics, Indianapolis, IN, USA), as described previously [34]. Hematology and biochemistry routine assays were performed at the accredited laboratory at Oslo University Hospital, Norway.

### Whole blood gene expression

In a subgroup of 29 CFS patients and 18 healthy controls, RNA was extracted from whole blood as described in details elsewhere [13]. RNA samples with RNA integrity number value ≥ 7 were used for gene expression characterization by RNA sequencing (RNA-Seq) (Run by the Genomics Core Facilities at the Oslo University Hospital Radiumhospitalet). RNA reads were mapped to the human genome version GRCh38.p2 by STAR [37]. Gene expression abundance was quantified as reads mapped per exon by the Subread package [38]. Differentially expressed genes (DEG) between CFS patients and controls were identified using DESeq2 package [39]. In order to correct for possible confounding background factors, age groups as scaling factor and gender input were included in the design model of DESeq2. Functional annotation of genes obtained from DESeq2 was done by uploading all DEGs into HumanMine [40 ].

### Clinical markers

A CFS symptom inventory for adolescents assesses the frequency of 24 common symptoms during the preceding month, as has been described elsewhere [34]. Briefly, each symptom is rated on a 5-point Likert scale, ranging from ‘never/rarely present’ to ‘present all the time’. The inventory includes the accompanying symptom of the Fukuda and Canada 2003 case definitions, facilitating post hoc subgrouping of CFS patients (Additional file 1). A composite score reflecting inflammatory symptoms was generated by taking the arithmetic mean across three single items (fever/chills, sore throat, and tender lymphatic nodes) and a composite score reflecting symptoms of post-exertional malaise was generated by taking the arithmetic mean across two single items (post-exertional fatigue and non-refreshing sleep). For both variables, the total range is from 0 to 5; higher scores imply more severe symptom burden.

The Chalder Fatigue Questionnaire (CFQ) total sum score is applied in the present study [41]; total range is from 0 to 33, where higher scores imply more severe fatigue. The mood and feelings questionnaire (MFQ) consists of 34 items, each scored on a 0–2 Likert scale; thus, the total sum score is from 0 to 68 [42]. The Spielberger State-Trait Anxiety Inventory subscore reflecting trait anxiety is derived from the sum across 20 items; total range is from 20 to 80 [43]. The *activPAL* accelerometer device (PAL Technologies Ltd, Glasgow, Scotland) was used for monitoring of daily physical activity during seven consecutive days [44], as described elsewhere [34].

### Statistical analyses

All statistical analyses were carried out in SPSS (SPSS Inc., Chicago, Illinois, USA). A total of 9 individuals (6 CFS patients, 3 healthy controls) had missing data for TGF-β analyses. As the CFS patients were included in a randomised controlled trial, individual data from follow-up consultations (when available) were used for imputation of missing data at baseline. The remaining missing data (3 CFS patients, 3 healthy controls) were imputed using the mean values within each group. Missing data in other variables were not imputed.

Patients with CFS were compared with healthy controls by applying independent sample t tests, Mann–Whitney tests, Pearson Chi Square tests, or Fisher exact tests as appropriate. CFS patients adhering to the Fukuda criteria and the Canada 2003-criteria were compared to the healthy controls in the same way. Associations between TGF-β and immune markers, neuroendocrine markers, single gene transcriptional counts and clinical markers within each group (CFS patients and healthy controls, respectively) were performed using correlation and linear regression analyses. A p < 0.05 was regarded as statistically significant. The correlation and linear regression analyses involved a multitude of statistical tests; however, as all these tests were considered exploratory, the p values were not adjusted.

## Results

### Demographic data

The 120 CFS patients fulfilling the inclusion criteria in the NorCAPITAL trial were included as the CFS patients group and 68 healthy individuals were included as control group [34]. The whole blood gene expression analysis (RNA-seq) was carried out in a random subgroup of 29 CFS patients and 18 healthy controls [13]. Gender, age and body mass index were equally distributed in the two groups (Table 1). In the CFS group, 98% were of Scandinavian ethnicity; 73% adhered to the Fukuda case definition and 38% to the Canada 2003-definition. With the exception of 1 patient, all belonging to the Canada 2003 subgroup also belonged to the Fukuda subgroup. A total of 2 CFS patients were on thyroid hormone supplement therapy, 1 used melatonin and 1 used bronchodilators occasionally; no other pharmaceuticals were used. No CFS patients and 3 healthy controls reported occasional use of illicit drugs; 78% in both groups reported no consume of alcoholic beverages. CFS patient had higher levels of serum CRP, plasma norepinephrine and plasma epinephrine, and lower levels of heart rate variability (HRV) LF-power and urine cortisol-to-creatinine ratio (Table 1). Also, CFS patients scored significantly poorer on all clinical markers (Table 1).

**Table 1:** Demographic characteristics

	CFS patients (n = 120)		Healthy controls (n = 68)		p value^c^
Background					
Female gender Number, %	88	73	46	68	0.563
Age (years) Mean, SD	15.4	1.6	15.1	1.6	0.179
Body mass index (kg/m^2^) Mean, SD	21.5	4.2	20.6	3.7	0.119
Disease duration (months) Median, range	18	4–104	n/a		
Adheres to the Fukuda criteria of CFS^a^ Number, %	88	73	n/a		
Adheres to the Canada 2003-criteria of CFS^b^ Number, %	46	38	n/a		
Immune, autonomic and neuroendocrine markers					
Serum C-reactive protein (mg/l) Median, IQR	0.43	0.96	0.35	0.46	*0.049*
Heart rate variability, LF power (abs) Median, IQR	481	772	682	1514	*0.015*
Heart rate variability, HF power (abs) Median, IQR	788	1835	1058	1837	0.070
Heart rate variability, LF/HF-ratio Median, IQR	0.69	0.64	0.74	0.38	0.792
Plasma norepinephrine (pmol/l) Mean, SD	1991	783	1483	422	*<* *0.001*
Plasma epinephrine (pmol/l) Median, IQR	308	131	265	95	*0.001*
Plasma cortisol (nmol/l) Mean, SD	367	143	351	150	0.460
Urine norepinephrine/creatinine ratio (nmol/mmol) Median, IQR	12.3	5.8	10.8	5.8	0.102
Urine epinephrine/creatinine ratio (nmol/mmol) Median, IQR	1.3	1.2	1.5	1.0	0.649
Urine cortisol/creatinine ratio (nmol/mmol) Median, IQR	3.4	3.1	5.3	6.0	*0.001*
Clinical makers					
Symptoms of post-exertional malaise (total score) Mean, SD	4.0	1.0	1.4	0.5	*<* *0.001*
Inflammatory symptoms (total score) Mean, SD	2.1	0.9	1.3	0.5	*<* *0.001*
Chalder Fatigue Questionnaire (total score) Mean, SD	19.3	6.1	8.7	4.6	*<* *0.001*
Moods and Feelings Questionnaire (total score) Mean, SD	17.4	10.0	6.4	7.8	*<* *0.001*
Spielberger state-trait anxiety questionnaire (trait subscore) Mean, SD	43.2	8.9	32.3	7.2	*<* *0.001*
Steps per day (number) Mean, SD	4662	2386	10,293	3716	*<* *0.001*

P values in italics indicate statistical significance

*n/a* not applicable, *SD* standard deviation, *IQR* interquartile range, *LF* low-frequency power of heart rate variability, *HF* high-frequency power of heart rate variability

^a^Cf. Ref. [35]

^b^Cf. Ref. [33]

^c^Statistical comparison across CFS group and HC group are based upon Independent sample t test, Mann–Whitney test or Fisher exact test as appropriate

### Transforming growth factor-β (TGF-β)

Plasma levels of all three isoforms of TGF-β (TGF-β1, TGF-β2 and TGF-β3) were equal in CFS patients and healthy controls (Table 2, Additional file 2: Table S1). Subgroup analyses revealed no differential results, neither for the Fukuda case definition nor for the Canada 2003-definition. Within the CFS group, TGF-β was not associated with clinical markers (Additional file 3: Table S2). Also, whole blood transcriptional counts from the *TGFB1* gene in the subgroup subjected to RNA-seq were equal among CFS patients and healthy controls (1184 vs 1208, p = 0.712).

**Table 2:** TGF-β isoforms across CFS patients (including Fukuda and Canada 2003-subgroups) and healthy controls

	CFS patients (n = 120)		p value^a^	Healthy controls (n = 68)		CFS, Fukuda subgroup (n = 88)		p value^a^	CFS, Canada 2003 subgroup (n = 46)		p value^a^
Plasma TGF-β1 (pg/ml) Median, IQR	4927	5157	0.482	5692	6350	4944	4276	0.599	4880	6247	0.831
Plasma TGF-β2 (pg/ml) Mean, SD	758	262	0.928	754	278	712	246	0.809	780	252	0.621
Plasma TGF-β3 (pg/ml) Median, IQR	230	227	0.901	251	250	230	208	0.990	243	254	0.760

*TGF* transforming growth factor beta, *SD* standard deviation, *IQR* interquartile range

^a^Compared with healthy controls. Statistical comparisons across groups are based upon Independent sample t test or Mann–Whitney test as appropriate

### TGF-β, neuroendocrine markers and fatigue score

**Fig. 1:**
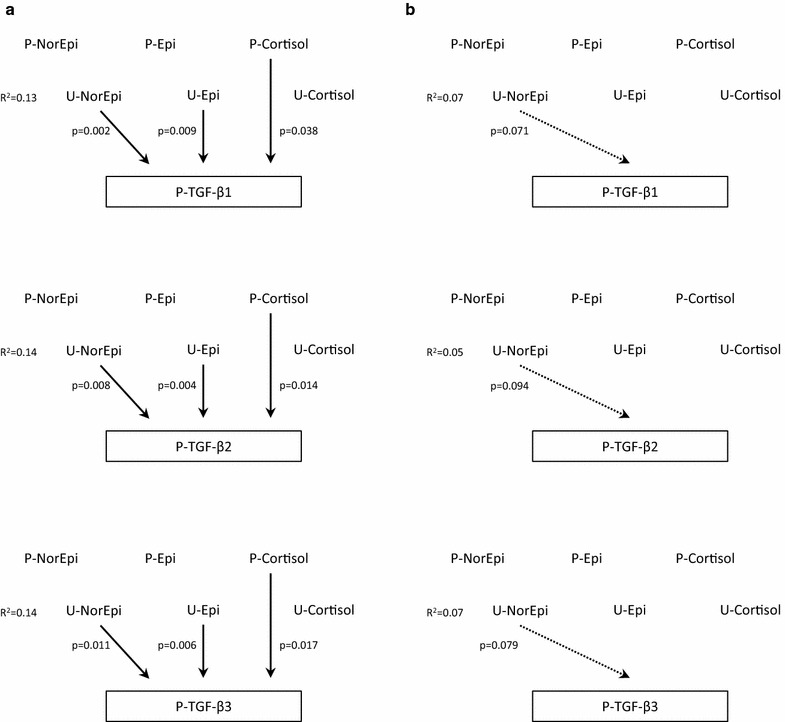
Multiple linear regression models exploring associations between neuroendocrine markers and isoforms of TGF-β in CFS patients (**a**) and healthy controls (**b**). Significant associations are indicated with a continuous arrow, whereas associations close to the level of significance are indicated with a dotted arrow. *P-NorEpi* plasma norepinephrine, *P-Epi* plasma epinephrine, *P-Cortisol* plasma cortisol, *U-NorEpi* urine norepinephrine-to-creatinine ratio, *U-Epi* urine epinephrine:creatinine ratio, *U-Cortisol* urine cortisol:creatinine ratio, *P-TGF* plasma transforming growth factor

Plasma levels of TFG-β were associated with neuroendocrine markers, in particular plasma cortisol as well as plasma and urine catecholamines. Multiple linear regression analyses revealed that these associations differed in CFS patients and healthy controls (Fig. 1), despite similar levels of TFG-β in the two groups. In the CFS patients, all TGF-β isoforms were significantly and independently associated with plasma cortisol, urine norepinephrine-to-creatinine ratio and urine epinephrine-to-creatinine ratio, whereas no significant associations to these markers were observed among healthy control. This differential association pattern was related to symptom severity (Table 3). When divided according to fatigue severity (Chalder Fatigue Questionnaire), CFS patients with the highest fatigue score (≥ 20) displayed the strongest associations between TGF-β and neuroendocrine markers, whereas CFS patients with the lowest fatigue score (< 20) had a pattern of associations that was more similar to the one observed in healthy controls.

**Table 3:** Multiple linear regression models for neuroendocrine markers and plasma TGF-β in CFS patients with high fatigue score (≥ 20), CFS patients with low fatigue score (< 20), and healthy controls

	CFS patients, high fatigue score (n = 62)^b^			CFS patients, low fatigue score (n = 54)^b^			Healthy controls (n = 68)		
Model 1									
Dependent variable									
Plasma TGF-β1^a^	B (CI)	p value	R^2^	B (CI)	p value	R^2^	B (CI)	p value	R^2^
Independent variables									
Plasma cortisol	1.9 × 10^−3^ (0.18 to 3.5 × 10^−3^)	*0.031*	0.17	0.26 × 10^−3^ (− 1.6 to 2.1 x 10^−3^)	0.780	0.12	− 0.19 × 10^−3^ (− 1.9 to 1.5 × 10^−3^)	0.825	0.07
Urine norepinephrine/creatinine ratio^a^	0.99 (0.20 to 1.8)	*0.015*		0.60 (0.01 to 1.2)	*0.048*		0.62 (− 0.06 to 1.3)	0.071	
Urine epinephrine/creatinine ratio^a^	− 0.65 (− 0.65 to − 0.06)	*0.031*		− 0.39 (− 0.77 to − 0.02)	*0.042*		0.004 (− 0.47 to 0.48)	0.987	
Model 2									
Dependent variable									
Plasma TGF-β2	B (CI)	p value	R^2^	B (CI)	p value	R^2^	B (CI)	p value	R^2^
Independent variables									
Plasma cortisol	0.64 (0.22 to 1.1)	*0.003*	0.22	0.07 (− 0.49 to 0.62)	0.814	0.12	− 0.01 (− 0.49 to 0.46)	0.950	0.05
Urine norepinephrine/creatinine ratio^a^	248 (50 to 447)	*0.015*		133 (− 44 to 311)	0.136		160 (− 28 to 349)	0.094	
Urine epinephrine/creatinine ratio^a^	− 161 (− 308 to − 14)	*0.032*		− 132 (− 246 to − 18)	*0.024*		− 16 (− 148 to 115)	0.806	
Model 3									
Dependent variable									
Plasma TGF-β3^a^	B (CI)	p value	R^2^	B (CI)	p value	R^2^	B (CI)	p value	R^2^
Independent variables									
Plasma cortisol	2.0 × 10^−3^ (0.60 to 3.4 × 10^−3^)	*0.006*	0.20	0.17 × 10^−3^ (− 1.6 to 1.9 × 10^−3^)	0.851	0.11	− 0.4 × 10^−3^ (− 1.8 to 0.99 × 10^−3^)	0.565	0.07
Urine norepinephrine/creatinine ratio^a^	0.78 (0.12 to 1.4)	*0.022*		0.40 (− 0.17 to 1.0)	0.164		0.50 (− 0.06 to 1.1)	0.076	
Urine epinephrine/creatinine ratio^a^	− 0.52 (− 1.0 to − 0.03)	*0.038*		− 0.41 (− 0.77 to − 0.05)	*0.027*		− 0.01 (− 0.40 to 0.38)	0.964	

P values in italics indicate statistical significance

*B* regression coefficient (unstandardized), *CI* confidence interval, *R* ^*2*^ explained variance of the dependent variable

^a^The ln-transformed variable was used (better approximation to a normal distribution)

^b^Fatigue score was missing from 4 CFS patients

### Immune gene transcripts

**Fig. 2:**
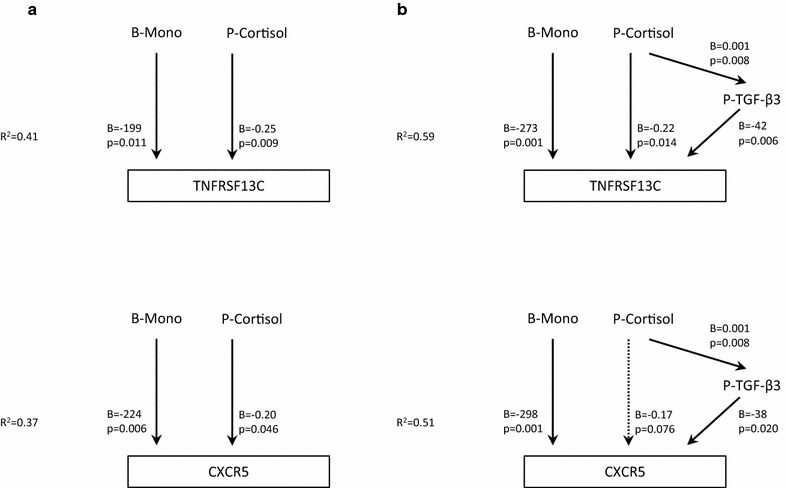
Multiple linear regression models in a subgroup of CFS patients (n = 29). **a** Previous established associations between blood monocyte count and plasma cortisol, and the expression in whole blood of the genes *TNFRSF13C* (tumor necrosis factor receptor superfamily member 13C) and *CXCR5* (C-X-C motif chemokine receptor 5) (Nguyen et al. [13]). **b** The associations after introducing TGF-β3 as a mediating variable in the regression model. Significant associations are indicated with a continuous arrow, associations close to the level of significance are indicated with a dotted arrow. *B-Mono* blood monocyte count, *P-Cortisol* plasma cortisol, *P-TGF* plasma transforming growth factor

Only a few differentially expressed immune gene transcripts were associated with TGF-β (Additional file 4: Table S3). However, there was a negative association, most evident for the TGF-β3 isoform, to the B cell annotated genes *TNFRSF13C* and *CXCR5*, which are both downregulated in CFS. Multiple linear regression analyses were consistent with a model in which TGF-β3 partly mediated the effect of plasma cortisol on the expression of these genes (Fig. 2).

## Discussion

There were three main finding of this study: (1) plasma levels of the three TGF-β isoforms did not differ in adolescent CFS patients as compared to healthy controls. (2) Within the CFS group, plasma levels of all TGF-β isoforms were associated with plasma levels of cortisol and urine levels of catecholamines. In addition, TGF-β3 might partly mediate the association between plasma cortisol and downregulation of some B cell annotated genes. (3) Plasma level of TGF-β was not directly related to CFS symptoms; however, the association of neuroendocrine mediators with TGF-β were related to fatigue score.

The lack of differences between CFS patients and healthy controls corroborate previous findings of normal plasma cytokine levels in CFS [17, 18]. Of note, subgrouping according to the Fukuda criteria or the Canada 2003-criteria did not reveal differential results. This is also in line with previous observations [17, 45]. While immunological alterations have been reported in many CFS studies, there seems to be no consistent pattern of plasma cytokine disturbances, calling for more specific investigations of immune mechanisms in different bodily compartments.

Previous studies have demonstrated a complex and possible bi-directional relationship between TGF-β levels and HPA axis activity [32, 46]. Similarly, TGF-β secretion has been associated with sympathetic nervous activity [47, 48] as well as plasma catecholamines [49]. Interestingly, in the present study, linear associations between TGF-β levels and neuroendocrine markers were found among CFS patients only. Moreover, within the CFS group, this picture was remarkably consistent across all TGF-β isoforms (Fig. 1), and related to the clinical symptom of fatigue (Table 3). Taken together, the results of the present study seem to suggest that underlying disease mechanisms of CFS are more related to altered immunological *control* than to altered levels of immune markers per se. Similar observations have been reported in a study of neuroendocrine regulatory systems [29], and are also in line with findings of a relationship between HPA axis dynamics and clinical symptoms [50, 51].

On a more general level, the findings of the present study seem to comply with the “sustained arousal” model of CFS [30]. In this model, a maladaptive stress response is considered a central pathophysiological element, eliciting autonomic and neuroendocrine alterations which in turn are responsible for subtle immunological alterations. Thus, an association between neuroendocrine markers and immune markers is predicted from this model, in line with the reported findings. It should be noted, though, that the cross-sectional design of the present study does not allow inference of causality. Further research should aim at uncover underlying mechanisms; one promising field of study might be epigenetic alterations of the glucocorticoid receptor gene [52].

The findings of a possible relationship between TGF-β3 and B cell annotated gene transcripts should be interpreted with great caution due to the limited number of CFS patients undergoing RNA-seq (n = 29). However, the findings were rather specific for the TGF-β3 isoform, reducing the risk of a type 1-error. Besides, it is not biologically implausible that TGF-β3 might act as a mediator of a cortisol effect on B cell gene expression. This observation should be explored further in mechanistic studies of immune cell function, in particular since the Epstein–Barr virus (EBV) is known to be a common precipitating factor in CFS [1]. Infection of EBV in human is persistent and tightly associated with B cell differentiation pathways. EBV establishes its long-term latency in memory B cells where its ability of switching from latent to lytic form of infection is related to the differentiation state of the cell [53]. Interestingly, it has been shown that TGF-β can induce EBV reactivation in vitro [54], and that expression of some of EBV promoters is activated in B cells upon TGF-β binding to TGF receptors [55].

In general, however, our data indicate that all three isoforms of TFG-β behave similar and that the previous data published with TFG-β, using isoform 1, is transferable to the other two isoform. To the best of our knowledge, this is the first study comparing the three TGF-isotypes. Of even more importance is that the only cytokine from the meta-analysis of 38 articles and 77 cytokines [18] shown to be a possible mediator discriminating CFS patients from controls, namely the TGF-β, fail to be shown in the present study, leaving no systemic cytokines left for this purpose.

Limitations of the present study encompass the wide inclusion criteria which might have obscured results pertaining to a subgroup. However, adjusting for adherence to the Fukuda and Canada 2003 case definitions did not alter the cross-sectional comparison. Also, the fact that our inclusion criteria are not identical to common international standards might reduce generalizability. As this study only included adolescents, we do not know to what extent the results apply to adult CFS patients. The number of patients that underwent whole blood gene expression by RNA-seq was rather low, increasing the risk of type II-errors. Although blood samples for cortisol analyses were obtained at a standardized time point, we cannot rule out that sleep problems and disturbances of the diurnal rhythm in the CFS group might have confounded the results. The composite scores reflecting inflammatory symptoms and symptoms of postexertional malaise has not been formally validated, and it is possible that a relationship between TGF-β levels and symptoms might have been discovered using inventories with higher sensitivity. Furthermore, we have not assessed responses to exercise and other stimuli. Normal levels at rest do not rule out abnormal response patterns. Finally, the nature of a cross-sectional study does not allow any causal inferences to be made.

## Conclusions

The main finding of this study is that plasma levels of the three TGF-β isoforms were equal in a large group of adolescent CFS patients compared to healthy controls. In addition, within the CFS group, plasma level of all TGF-β isoforms were associated with plasma levels of cortisol and urine levels of catecholamines, and these associations were related to fatigue score. Finally TGF-β3 might partly mediate an association between plasma cortisol and downregulation of some B cell annotated genes. These latter observations should be addressed in further studies aiming at unraveling the complex causal interplays between neuroendocrine signaling, subtle immune alterations and clinical symptoms in CFS.

## Additional files

**Additional file 1.** Methods for post hoc-subgrouping of patients in the NorCAPITAL project according to the Canada 2003 and Fukuda case definitions.

**Additional file 2: Table S1.** Plasma levels of TGF-β1, TGF-β2, TGF-β3 in individual CFS patients and healthy controls (raw data).

**Additional file 3: Table S2.** Correlations (Pearson’s r) between the three isoforms of TGF-β and immune markers, neuroendocrine markers and clinical markers in CFS patients and healthy controls.

**Additional file 4: Table S3.** Correlations (Pearson’s r) between the three isoforms of TGF-β and immune annotated single gene transcriptional counts in a subgroup of CFS patients (n = 29).

## Abbreviations

CFS: chronic fatigue syndrome
TGF: transforming growth factor
NorCAPITAL: The Norwegian Study of Chronic Fatigue Syndrome in Adolescents: Pathophysiology and Intervention Trial
HPA axis: hypothalamus–pituitary–adrenal axis
IL: interleukin
TFN: tumor necrosis factor
CRP: C-reactive protein
RNA-Seq: RNA sequencing
DEG: differentially expressed genes
HPLC: high-performance liquid chromatography
HRV: heart rate variability
LF: low frequency
HF: high frequency
CFQ: Chalder Fatigue Questionnaire
MFQ: Moods and Feelings Questionnaire
